# Trends in Asthma-Related Direct Medical Costs from 2002 to 2007 in British Columbia, Canada: A Population Based-Cohort Study

**DOI:** 10.1371/journal.pone.0050949

**Published:** 2012-12-05

**Authors:** Pierrick Bedouch, Carlo A. Marra, J. Mark FitzGerald, Larry D. Lynd, Mohsen Sadatsafavi

**Affiliations:** 1 Collaboration for Outcomes Research and Evaluation, Faculty of Pharmaceutical Sciences, University of British Columbia, Vancouver, British Columbia, Canada; 2 Center for Clinical Epidemiology and Evaluation, Vancouver Coastal Health Institute, Vancouver, British Columbia, Canada; 3 Division of Respiratory Medicine, Faculty of Medicine, University of British Columbia, Vancouver, British Columbia, Canada; Public Health Agency of Barcelona, Spain

## Abstract

**Background:**

Asthma-related health resource use and costs may be influenced by increasing asthma prevalence, changes to asthma management guidelines, and new medications over the last decade. The objective of this work was to analyze direct asthma-related medical costs, and trends in total and per-patient costs of hospitalizations, physician visits, and medications.

**Methods:**

A cohort of asthma patients from British Columbia (BC), Canada, was created. Asthma patients were identified using a validated case definition. Costs for hospitalizations, physician visits, and medications were calculated from billing records (in 2008 Canadian dollars). Trends in total and per-patient costs over the study period were analyzed using Generalized Linear Models.

**Results:**

398,235 patients satisfied the asthma case definition (mid-point prevalence 8.0%). Patients consumed $315.9 million (M) in direct asthma-related health resources between 2002 and 2007. Hospitalizations, physician visits, and medication costs accounted for 16.0%, 15.7% and 68.2% of total costs, respectively. Cost of asthma increased from $49.4 M in 2002 to $54.7 M in 2007. Total annual costs attributable to hospitalizations and physician visits decreased (−39.8% and −25.5%, respectively; p<0.001), while medication costs increased (+38.7%; p<0.001).

**Interpretation:**

This population-based analysis shows that the total direct cost of asthma in BC has increased since 2002, mainly due to a rise in asthma prevalence and cost of medication. Combination therapy with inhaled corticosteroids/long-acting beta-agonists has become a significant component of the cost of asthma. Although billing records capture only a fraction of the true burden of asthma, the simultaneous increase in medication costs and reductions in hospitalization and physician visit costs provides valuable insight for policy makers into the shifts in asthma-related resource use.

## Introduction

Asthma is one of the most common chronic diseases in western countries [1], [2]. In Canada, 14.1% of the population is estimated to have been diagnosed with asthma at some time in their life [1], and there is evidence that the prevalence of asthma has increased in Canada and other western countries during the last decade [2]. For example, a population-based study conducted in Ontario, Canada, found that age- and sex-standardized asthma prevalence has increased by 55% between 1996 and 2005 [3]. However, there is also evidence from Canada of over-diagnosis of asthma, which would result in an over-estimation of prevalence [4].

The economic burden of asthma is significant in developed countries [2], [5], [6], and there is evidence that costs are highest among patients with suboptimal control of asthma [7]. In this context, the primary goal of asthma management is to control the disease to prevent morbidity and mortality in a cost-effective way. New guidelines aimed at improving asthma management, as well as new medications, have been introduced over the past ten years [8]–[12]. Nonetheless, between 47% and 82% of Canadians with asthma remain inadequately controlled [7], [13], [14].

British Columbia (BC) has a population of 4.5 million, representing approximately 13% of Canada’s total population [15]. The BC provincial health insurance program collects administrative data on the health resource use of all BC residents. The purpose of this population-based cohort study was to use BC administrative data to: 1) calculate the direct medical costs attributable to asthma in BC for the years 2002–2007; 2) evaluate asthma-related costs according to the level of asthma control; and 3) analyze trends in total and per-patient costs, and to evaluate trends in hospitalizations, physician visits, and medication costs, and level of asthma control, over this period.

## Methods

### Study Design and Setting

A population-based retrospective cohort study was conducted using the administrative health data of BC, which includes all BC residents registered in the province’s publicly funded universal insurance program. Population Data BC, a pan-provincial population health data service, provides data linkage, development, and access to the health services database [16]. Data for the fiscal years 1997 to 2007 were obtained (a fiscal year, referred to hereafter as ‘year’ in this report, runs from April 1 of one year to March 31 of the following year). The first five years were used as a ‘wash-in’ period in order to give sufficient time for the prevalent cases of asthma to be identified [3]; as such, all outcomes are reported for the 6-year period spanning from 2002 to 2007. Ethics approval was provided by the Clinical Research Ethics Board at the University of British Columbia.

### Data Source

The following components of health resource utilization were retrieved: 1) The Discharge Abstracts Database (DAD) for hospital separations; 2) Medical Services Plan (MSP), which includes records of physician visits; 3) The Vital Statistics database, which provides the date and cause of death; and 4) Demographics and Registration databases, which provide basic demographic information as well as longitudinal registration status in the provincial healthcare system. In addition, for each patient we retrieved data from the provincial PharmaNET system, which contains the quantity and days of supply for every prescription drug dispensed to members of the provincial health insurance program [17].

### Asthma Case Definition

A validated case-definition of asthma was used to identify all asthma patients in BC who used health care resources over the study period [18]. According to this case definition, patients were considered to have asthma if, during a 12-month rolling window, they had at least one asthma-related hospitalization (codes of the International Classification of Diseases-9^th^ edition (ICD-9): 493.x, 10^th^edition (ICD-10): J45, J46); two or more physician visits with diagnostic code of asthma (ICD-9:493.x); or filled four or more prescriptions for asthma-related medications (list of asthma-related medications provided in Table S1). In order to prevent cases of childhood respiratory diseases and cases of obstructive lung diseases from being falsely identified as asthma, patients who only satisfied the asthma case definition when they were younger than 5 years or older than 55 years were excluded if they had either a physician visit or a hospital discharge record with the most responsible diagnosis of childhood or obstructive lung diseases (Table S2).

Once a patient was determined to have asthma based on the case definition, a look-back algorithm was applied in which we explored the patient’s history of asthma-related resource use. The date of the patient’s first asthma-related resource use was used as the *index date* and all resource use after this date was considered. In the base-case analysis we included all patients regardless of age, consistent with a recent study of asthma using administrative data [3]. In a sensitivity analysis, only patients who satisfied the case definition criteria when they were between 5 and 55 years of age were included.

### Asthma-related Health Care Resource Utilization

In the base-case analysis, a narrow definition of asthma-related resource use was employed for all cost components. Hospitalizations included all hospital discharges for which asthma was the most responsible diagnosis. Physician encounters were defined as asthma-related if asthma was listed as the primary reason for the visit. For medication dispensations, we created a short, specific list of asthma-related medications. In a sensitivity analysis, a broader definition of asthma-related resource use was employed: all hospital records in which asthma was listed among the discharge diagnoses were defined as asthma-related (ICD-9: 493.x, ICD-10: J45, J46); all physician visits in which asthma or a related diagnosis (see Table S3) was recorded were defined as asthma-related; and for asthma-related medication use, we used a less-specific list of asthma-related drugs (Table S1).

In order to classify patients as being controlled or uncontrolled in each year of the study period, a previously published algorithm developed by Firoozi *et al*. was applied [19]. Based on resource use data in the past 12 months, this algorithm classifies each individual at a given time point into having controlled or uncontrolled asthma. Factors considered in this classification include the daily dose of inhaled corticosteroids (ICS), use of other asthma controller therapies (long-acting beta agonists [LABA], theophylline, and leukotriene receptor antagonists [LTRA]), weekly dose of short-acting beta-agonists (SABA), and the presence of markers of moderate-to severe exacerbations (hospital admission or ED visit due to asthma, or use of oral steroids). This algorithm was applied to each person-year of the data, allowing individuals to move between controlled and uncontrolled states over time. As the original algorithm was designed for patients 14 years and older, we performed this analysis using only patient-years in this age group.

### Calculations of Asthma-related Costs

Costs of hospitalizations, physician visits, and medications were calculated separately and then aggregated to provide the total direct cost of asthma-related resource use. In Canada, hospitalization records are maintained nationally by the Canadian Institute of Health Information (CIHI). This agency classifies each hospitalization record using Case Mix Group methodology, in which hospitalizations are categorized into statistically and clinically homogeneous groups based on the collection of clinical and administrative data [20], [21]. A resource intensity weight (RIW) is assigned to each hospitalization record based on the case mix group as well as the patient's demographic variables, lengths of stay, transfers, and so on. A RIW of 1 means the corresponding episode of hospitalization had an ‘expected’ cost equal to the average cost of a hospitalization period in that province in that fiscal year (Cost Per Weighted Case, CPWC) [22], [23]. To determine the cost of each hospitalization, we therefore multiplied the RIW by CPWC for the corresponding fiscal year. Because Emergency Department (ED) visits are not systematically recorded in any of BC’s administrative databases, costs associated with ED visits were imputed by assuming a constant rate of ED visits to hospitalizations based on previous Canadian studies [24], [25] and assigning a unit cost for an ED visit ($406 in 2008 Canadian dollars) [26]. ED visit costs were then added to the costs of hospitalizations. Costs of physician visits and medication use included both the cost covered by the insurer as well as out-of-pocket costs.

The total cost of asthma for a given year is the sum of all asthma-related patient-level costs. Asthma is still viewed as a disease which is at best controlled, but not cured, and that the state of control often changes considerably during the lifetime of patients. In this context, average cost per person-year can be defined in at least two logical ways: whether the interest is in the cost per patient-year for patients who have ever had asthma, or in the cost per patient-year for patients with current (or active) asthma. We considered both situations for calculating the number of patients with asthma in the province in any given year. In the first method, once a patient satisfied the asthma case definition, they contributed to the person-years with asthma for as long as they were registered in the provincial health program. In the second method, we only considered years in which at least one asthma-related resource use was recorded for the patient. All costs were reported in inflation-adjusted 2008 Canadian dollars ($) using the health and personal care component of the BC consumer price index [27]. Costs for all the population, as well as for the subgroups defined by sex and age were reported. Trends of total annual and per patient costs over the study period were analyzed using Generalized Linear Models with gamma distribution and logarithmic link function.

## Results

### Participants

From 2002 to 2007, 398,235 unique patients fulfilled the case definition of asthma (Table 1). The mean age at index date was 32.8 years (standard deviation 21.7). There were a total of 1,977,199 patient-years of follow-up, or, on average, 5.0 years of follow-up per patient. The prevalence of asthma increased monotonically from 7.1% in 2002 to 8.3% in 2007 (mid-point prevalence 8.0%). Table 2 presents the breakdown of health care resource use.

**Table 1:** Age, sex, and asthma prevalence of the study population (N = 398,235).

Characteristic	N	%
Age at the index date		
<5 yrs	36,325	(9.1)
5–9 yrs	40,603	(10.2)
10–14 yrs	34,205	(8.6)
15–39 yrs	138,045	(34.7)
40–69 yrs	124,586	(31.3)
≥70 yrs	24,471	(6.1)
Sex (Female)	219,773	(55.2)
Asthma patients (Prevalence, %)		
2002	290,343	(7.1)
2003	309,684	(7.5)
2004	325,554	(7.8)
2005	340,792	(8.1)
2006	351,233	(8.3)
2007	359,593	(8.3)

**Table 2:** Health care resource use, total and per patient (2002–2007).

Health care resource	Encounters	
	Total	Per patient-year
Hospitalization*	7,807	4.0†
Physician visits	1,518,582	0.79
Asthma-related medications	4,463,903	2.26

* *Including Emergency Department visits;*

† *Per 1,000 patients.*

### Asthma Total Direct Cost and per Patient-year Cost

Overall, the cohort was responsible for $315,263,177 in direct health care costs during the 6-year study period. In the base-case analysis, hospitalizations, physician visits, and medications accounted for 16.0%, 15.7% and 68.3% of asthma-related costs, respectively. ***Table 3*** shows the results of sensitivity and subgroup analyses. Total cost of asthma varied from $263.2 million (M) in the most conservative to $465.3 M in the least conservative estimate. Children and adolescents (14 years or less) contributed to the 19.1% of patient-years, and were responsible for 16.8% of total direct costs. 55.3% of total person-years belonged to women, while this group generated 56.0% of total costs.

**Table 3:** Total annual costs and annual costs per patient: results of the sensitivity analyses (2008 Canadian dollars).

Scenario	Patient-years	Annual direct costs(Average per-patient cost)			
		Hospital/ED	Physician visits	Medications	Total
Narrow definition, all age (Baseline)*	1,977,199	$50,534,680($25.6)	$49,615,260($25.1)	$215,113,237($108.8)	$315,263,177($159.4)
Narrow definition, active asthma‡, all age*	1,013,975	$50,534,680($49.8)	$49,615,260($48.9)	$215,113,237($212.1)	$315,263,177($310.9)
Narrow definition, 5–55 yrs*	1,650,214	$46,253,069($28.0)	$43,455,249($26.3)	$173,484,112($105.1)	$263,192,430($159.5)
Broad definition, all age†	1,977,199	$138,038,038($69.8)	$98,570,345($49.9)	$228,729,607($115.7)	$465,337,990($235.4)
Broad definition, active asthma§, all age†	1,013,975	$138,038,038($136.1)	$98,570,345($97.2)	$228,729,607($225.6)	$465,337,990($458.9)
Broad definition, 5–55 yrs†	1,650,214	$115,148,968($69.8)	$84,492,885($51.2)	$183,148,586($111.0)	$382,790,439($232.0)
Pediatric patients (≤14 y/o)	377,816	18,838,495($49.9)	13,128,811($34.7)	21,117,435($55.9)	53,084,741($140.5)
Adults population (>14 y/o)	1,599,383	31,696,185($19.8)	36,486,449($22.8)	193,995,802($121.3)	262,178,436($163.9)
Male population	883,753	22,280,511($25.2)	22,763,367($25.8)	93,430,815($105.7)	138,474,694($156.7)
Female population	1,093,446	28,254,169($25.8)	26,851,893($24.6)	121,682,422($111.3)	176,788,483($161.7)

* Narrow definition: hospitalizations in which asthma was coded as the ‘most responsible’ diagnosis (i.e., ICD-9 493.x or ICD-10 J45, J46), physician visits that were coded as asthma according to the ICD-9 code 493.x, short list of asthma-related medications;

† Broad definition: all hospitalizations in which asthma was indicated among the discharge diagnoses, all physician visits for an asthma-related diagnosis, long list of asthma-related medications;

‡ Patient-years with active asthma are the years in which the patient has consumed at least one asthma-related health care resource. (hospitalization, physician visit or asthma-related medication).

**Figure 1:**
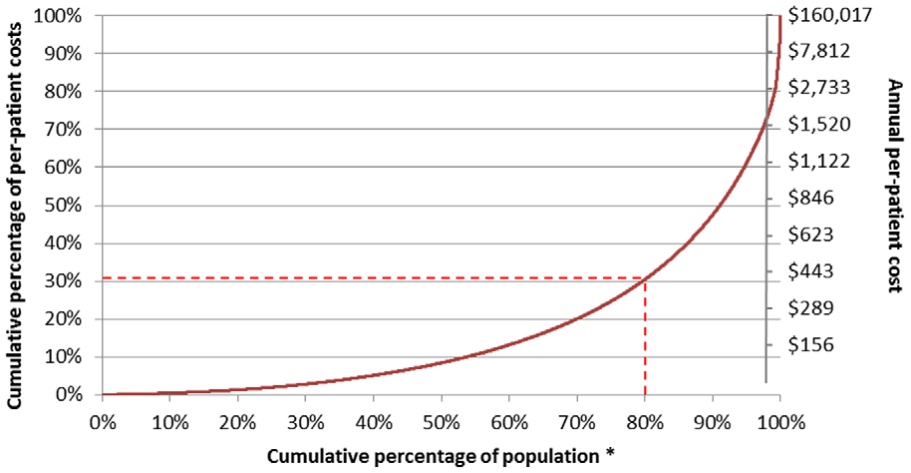
Cumulative distribution of per-patient costs according to the annual per-patient cost (right vertical axis) and the cumulative percentage of per-patient costs (left vertical axis) (2008 Canadian dollars). * Population = 1,977,199 patient-years of follow-up Dashes lines indicate that 80% of the cumulative percentage of population is responsible for 30.5% of the cumulative percentage of per-patient costs.

A total of 1,013,975 patient-years (51.3% of total patient-years) belonged to individuals who were categorized has having current asthma. The annual per-patient cost among patients with current asthma was $311, compared to $159 among all patients who ever had asthma. ***Figure 1*** presents the cumulative distribution of per-patient annual costs. Notably, 69.5% of the total costs were generated by the 20% of patient-years in which annual per-patient costs were higher than $450.

#### Trends over 2002–2007 in asthma control and direct asthma-related costs

**Figure 2:**
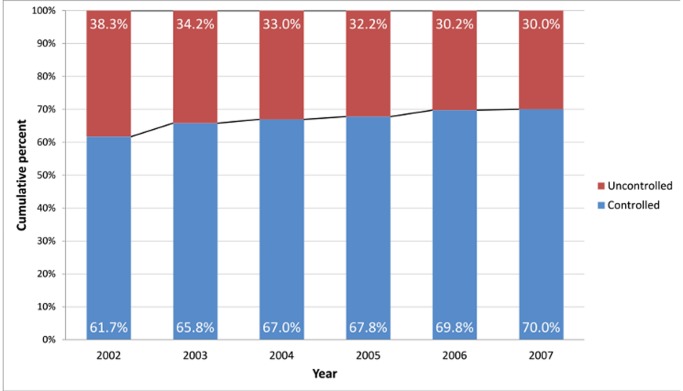
Trends over 2002–2007 in asthma control.

***Figure 2*** presents the trend in proportion of patient-years with controlled asthma to total patient-years with asthma for each year. Proportion of patient-years with controlled asthma increased consistently from 61.7% in 2002 to 70.0% in 2007.

**Figure 3:**
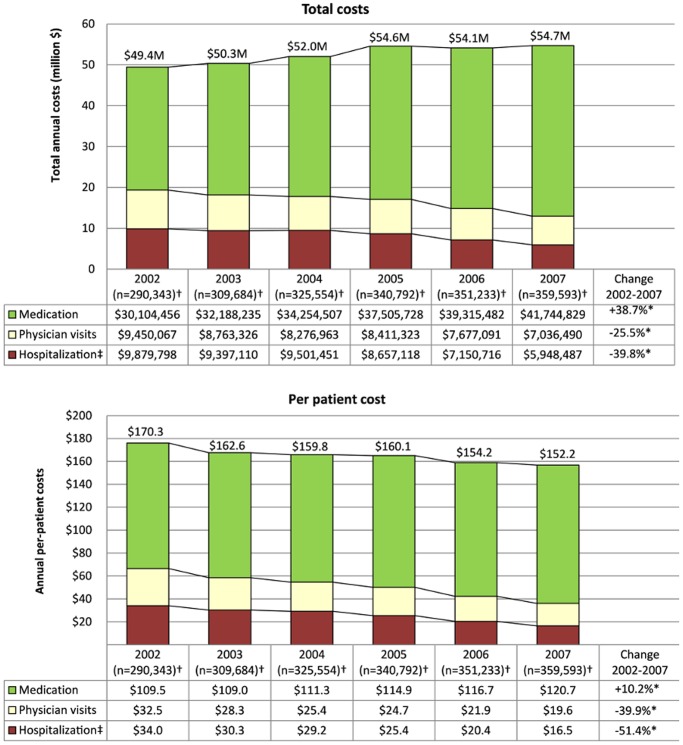
Trends over 2002–2007 in annual costs of asthma and cost component (2008 Canadian dollars). * P-value <0.001; † Number of patients for each year of data; ‡Including Emergency Department visits.

***Figure 3*** presents total asthma-related costs stratified by cost component and year. The annual total costs significantly increased by an additional 10.7% over the study period (p<0.001). Specifically, total annual costs increased from $49.4 M in 2002 to $54.6 M in 2005, reaching a plateau that remained stable until 2007 ($54.7 M). The proportion of costs attributable to each cost component shifted over time, with the cost of hospitalizations and physician visits decreasing by −39.8% (p<0.001) and −25.5% (p<0.001), respectively, while medication costs increased by +38.7% (p<0.001) over the study period, from $30.1 M in 2005 to $41.7 M in 2007.

Per-patient annual cost significantly decreased by 10.6% between 2002 and 2007 (p<0.001), corresponding to an average decrease of 2.3% per patient per year (p<0.001). This decrease was attributable to a reduction in the cost of hospitalizations and physician visits of 12.7% (p<0.001) and 9.2% (p<0.001) per patient per year, respectively. In contrast, the average per-patient annual asthma-related medication cost over the study period increased by 2.4% per year (p<0.001).

### Asthma-related Medication Cost by Drug Category

**Figure 4:**
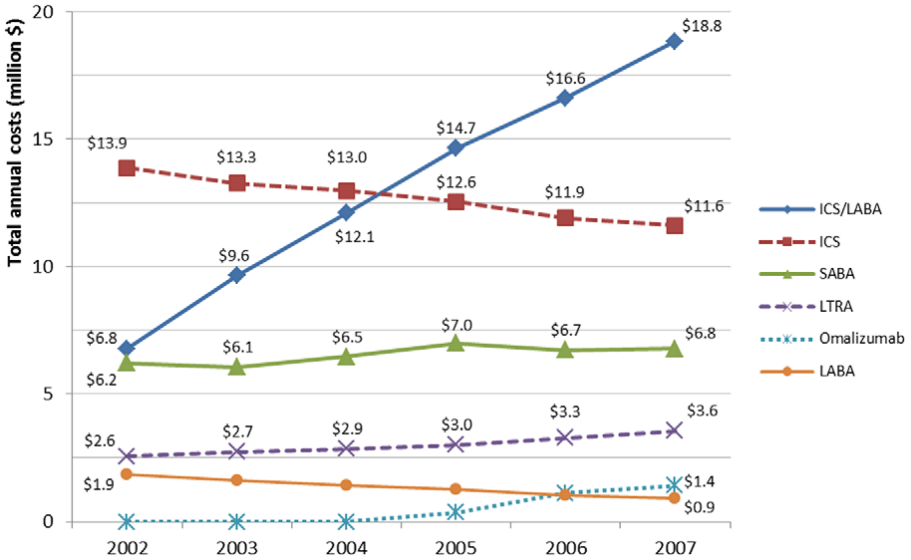
Annual cost of asthma-related medications according to year and drug category (2008 Canadian dollars). *ICS: inhaled corticosteroids, SABA: short-acting beta-agonists, LABA: long-acting beta-agonists, ICS/LABA: inhaled corticosteroids and long-acting beta-agonists in combination, LTRA: leukotriene receptor antagonists.*

The trend of medication costs by drug category over the period is presented in ***Figure 4***. The distribution of medication costs changed over the study period with a sharp increase in the cost of combined ICS and LABA (+178.0%) and LTRA (+38.3%). The cost of SABA showed slower growth (+9.0%) and the cost of ICS and LABA decreased by 16.3% and 49.7%, respectively, over the study period.

### Costs Stratified by Level of Asthma Control

**Figure 5:**
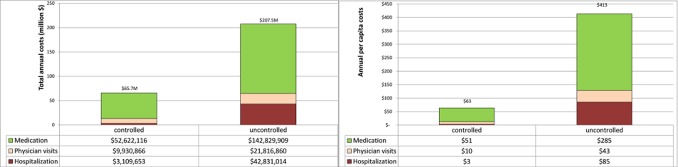
Total (left) and per-patient (right) cost of asthma according to the level of control from 2002 to 2007 (2008 Canadian dollars)* *for 293,055 patients over 14 years old at the index date.*

In total, 293,055 (73.6%) patients were 14 years or older at their index date, contributing 1,538,558 patient-years of data. Of these, 32.6% were classified as having inadequately controlled asthma. Uncontrolled asthmatics were responsible for $273.1 M, or 76.0% of the total costs of asthma care for patients in this age group. ***Figure 5*** shows the total (left panel) and per-patient (right panel) cost of asthma according to the level of control. All three components of costs were higher in patients considered to be uncontrolled. The ratio of annual costs per patient for uncontrolled vs. controlled asthma was 6.5 ($413 vs. $63).

## Discussion

This population-based analysis of all asthma patients registered in the BC provincial health insurance program shows that direct asthma-related health care costs in BC were $315.3 M between the years 2002 and 2007. Adjusting for population size and currency value, this estimated annual cost is somewhat lower than the direct costs of asthma calculated for the total population of Canada in 1990 ($435 M in 2008) [28]. Despite significant reductions in hospitalization and physician visit costs, the average annual cost of asthma increased approximately ten percent, as medication costs increased nearly forty percent from 2002 to 2007. The distribution of medication costs over the study period revealed a sharp increase in the cost of combination therapy with ICS/LABA (+178.0%). Notably, the cost of both ICS and LABA prescribed as mono-therapies decreased over the study period. This change is consistent with Canadian guidelines on asthma management, which recommend avoiding the use of LABA as mono-therapy and recommend the use of an ICS/LABA combination inhaler when both therapies are deemed necessary [12], [29]. Overall, the observed decline in the ratio of uncontrolled to controlled asthma, as well as the decline in the number of hospital admissions due to asthma and increase in controller medication use during the study period is consistent with the observed long-term trends [30].

To our knowledge, this is the first study to document a recent decrease in asthma-related hospitalizations and physician visit costs alongside an increase in medication costs. However, studies from other countries have shown decreases in asthma-related hospitalizations [31]–[33]. The average annual cost per patient of $159 estimated here could be considered low in comparison to annual per-patient costs ranging from US$616 to US$1,250, as found in studies using administrative databases in the United States (US) [34]–[37]. The discrepancies in these estimates likely reflect differences in cohort definitions, methods for attributing resource use to asthma, as well as true differences in costs between the US and Canada. However, differences in study designs, definitions of costs, and time periods make it difficult to compare our estimate of annual per-patient cost to those from other studies.

Some limitations must be considered when interpreting the results of this study. The algorithm used in this study to detect patients with asthma, although validated by others [18], inevitably results in the false exclusion of some asthma patients and the inclusion of non-asthmatics in the cohort; Specifically, this algorithm might have excluded patients with intermittent forms of asthma such as those with seasonal asthma, exercise asthma, and mild asthma cases that experience exacerbations after respiratory infections. However, we were concerned that a more lenient case definition would have unacceptably low level of specificity. Furthermore, in the absence of a control group from the same population, we did not attempt to calculate the incremental cost of asthma by comparing the total health care costs between asthmatics and controls. Rather, we adopted a conservative definition by explicitly attributing resource use records to asthma in calculating asthma-related costs. It is likely that additional co-morbidities associated with asthma were missed by our methods [38]. Based on administrative databases, our study did not capture certain direct medical costs, such as asthma education programs or peak flow meters, or indirect costs such as productivity losses. Given that asthma affects individuals of all age groups, the indirect cost of asthma can be substantial. In some studies, indirect cost of asthma has been found to exceed the direct costs [39], [40]. Asthma exacerbations are responsible for work and school day losses for patients and parents and/or caregivers of children with asthma, while occupational asthma is responsible for a considerable proportion of workers’ compensation claims [26], [28], [41]. The analysis of the distribution of average per-patient costs and the trend over time could have been performed using zero-inflated mixed models to properly account for variable cost-per patients and excess zeros in the data and within-patient correlation of annual costs; but given the large sample of the study, such analysis proved computationally infeasible.

Our study was not designed to discern any causal relationship between medication use and clinical outcomes, and many non-pharmacologic factors may have contributed to decreased hospitalization and physician visits costs among asthma patients. The implementation of patient education programs, self-management plans, and better outpatient case management could have reduced asthma exacerbations and the associated health resource use.

This study has provided a detailed description of asthma-related health resource use and costs over a six-year period and indicates that combination therapy with ICS/LABA has become the most significant cost of asthma management in BC. The trend analysis, showing an increase in medication costs compensated by a reduction in hospitalization and physician visit costs, provides valuable insight for policy makers into shifts in asthma-related resource use. Policy makers must be aware of the dynamic changes in the extent and pattern of the burden of asthma and the need for updating the evidence base given the noticeable trends in asthma-related recourse use and outcomes. Further research that examines the causal relationship between changes in medication prescribing patterns and the decline in asthma hospitalizations and physician visits will be of interest to both clinicians and policy makers.

## Supporting Information

Table S1 Asthma-related medications per Categories, Active ingredients, Anatomical Therapeutic Chemical (ATC) codes and Drug Identification Numbers (DIN) selected in the PharmaNet database
(DOCX)

Table S2 Exclusion of childhood and obstructive lung diseases among patients younger than 5 or older than 55 years old, International Classification of Diseases – Ninth Revision (ICD-9) and Tenth Revision (ICD-10) codes selected in the Discharge Abstracts Database (DAD)
(DOCX)

Table S3 Physician visits for asthma and asthma-related diagnoses used for the ‘broad definition’ of asthma-related resource use, International Classification of Diseases – Ninth Revision (ICD-9) codes selected in the Medical Service Plan (MSP) database
(DOCX)
