# Correction: Trends in Asthma-Related Direct Medical Costs from 2002 to 2007 in British Columbia, Canada: A Population Based-Cohort Study

**DOI:** 10.1371/annotation/a3275f00-6d75-4430-a0d6-5b4397ba501a

**Published:** 2013-06-18

**Authors:** Pierrick Bedouch, Mohsen Sadatsafavi, Carlo A. Marra, J. Mark FitzGerald, Larry D. Lynd

The authors are listed out of order. The correct author order and citation are:

Pierrick Bedouch, Mohsen Sadatsafavi, Carlo A. Marra, J. Mark FitzGerald, Larry D. Lynd

Bedouch P, Sadatsafavi M, Marra CA, FitzGerald JM, Lynd LD (2012) Trends in Asthma-Related Direct Medical Costs from 2002 to 2007 in British Columbia, Canada: A Population Based-Cohort Study. PLoS ONE 7(12): e50949. doi:10.1371/journal.pone.0050949 

