# Supplementary material for: Trends in Asthma-Related Direct Medical Costs from 2002 to 2007 in British Columbia, Canada: A Population Based-Cohort Study
Source: PLoS One. 2012 Dec 5;7(12):e50949. doi: 10.1371/journal.pone.0050949 (PMC3515523; doi:10.1371/journal.pone.0050949)
Supplement: Table S1 — Asthma-related medications per Categories, Active ingredients, Anatomical Therapeutic Chemical (ATC) codes and Drug Identification Numbers (DIN) selected in the PharmaNet database (DOCX) [file pone.0050949.s001.docx]

**Appendix Table 1:** Asthma-related medications per Categories, Active ingredients, Anatomical Therapeutic Chemical (ATC) codes and Drug Identification Numbers (DIN) selected in the PharmaNet database

| **Medication categories** | **Active ingredient(s)** | **ATC** | **DIN** |
| --- | --- | --- | --- |
| **Used in the ‘narrow definition’ of resource use (short list), also used for case-definition of asthma (see text)** | | | |
| Inhaled corticosteroids (ICS) | Beclometasone | R03BA01 | 2242030, 2242029, 374407, 828521, 828548, 872334, 893633, 897353, 1949993, 1950002, 2079976, 2213710, 2213729, 2215039, 2215047, 2215055, 2216531 |
|  | Budesonide | R03BA02 | 2229099, 1978918, 1978926, 852074, 851752, 851760 |
|  | Fluticasone | R03BA05 | 2237247, 2237246, 2237245, 2237244, 2244293, 2244292, 2244291, 2174731, 2174758, 2174766, 2174774, 2213583, 2213591, 2213605, 2213613 |
|  | Ciclesonide | R03BA08 | 2285614, 2285606, 2303671 |
| Short-acting beta-agonists (SABA) | Salbutamol | R03AC02 | 790419, 812463, 832758, 832766, 851841, 860808, 867179, 897345, 1926934, 1938851, 1938878, 1945203, 1947222, 1986864, 2022125, 2046741, 2048760, 2069571, 2084333, 2148617, 2154412, 2173360, 2208229, 2208237, 2208245 2212315, 2212323, 2213400, 2213419, 2213427, 2213478, 2213486, 2214997, 2215004, 2215616, 2215624, 2215632, 2216949, 2231430, 2231488, 2231678, 2231783, 2231784, 2232570, 2232987, 2236931, 2236932, 2236933, 2239365, 2239366, 2241497, 2243115, 2243828, 2244914, 2245669, 2259583, 2326450 |
|  |  | R03CC02 | 620955, 620963, 874086, 894249, 894257, 1932691, 2035421, 2063689, 2091186, 2146843, 2146851, 2164434, 2164442, 2165368, 2165376, 2212390, 2213435, 2213443, 2213451, 2261324 |
|  | Terbutaline | R03AC03 | 786616 |
|  | Orciprenaline | R03CB03 | 249920, 3891, 2236783, 2229862, 2152568, 2192675 |
| Long-acting beta-agonists (LABA) | Salmeterol | R03AC12 | 2211742, 2214261, 2231129, 2136139, 2136147 |
|  | Formoterol | R03AC13 | 2230898, 2237224, 2237225 |
| ICS and LABA in combination (ICS/LABA) | Budesonide, formoterol | R03AK07 | 2245385, 2245386 |
|  | Fluticasone, salmeterol | R03AK06 | 2240835, 2245126, 2245127, 2240836, 2240837 |
| Leukotriene receptor antagonists (LTRA) | Montelukast | R03DC03 | 2247997, 2238217, 2243602, 2238216 |
|  | Zafirlukast | R03DC01 | 2236606 |
| Anti-immunoglobulin E monoclonal antibody | Omalizumab | R03DX05 | 2260565 |
| Inhaled mast cell stabilizers | Cromoglicic acid | R03BC01 | 2231431, 2231671, 2046113, 534609, 555649, 261238, 638641, 2049082, 2219468 |
| Theophylline | Choline theophyllinate | R03DA02 | 346071, 405310, 441724, 441732, 451282, 458708, 458716, 476366, 476390, 476412, 503436, 511692, 536709, 565377, 589942, 589950, 792934 |
|  | Theophylline | R03DA04 | 156701, 261203, 460982, 460990, 461008, 466409, 488070, 532223, 556742, 575151, 599905, 627410, 631698, 631701, 692689, 692697, 692700, 722065, 1926586, 1926594, 1926608, 1926616, 1926640, 1966219, 1966227, 1966235, 1966243, 1966251, 1966278, 1966286, 2014165, 2014181, 2230085, 2230086, 2230087 |
|  | Aminophylline | R03DA05 | 14923, 178497, 497193, 497193, 497207, 582654, 582662, 868450, 2014270, 2014289 |
| **Additional medications used in the ‘broad definition’ of resource use (long list)** | | | |
| Inhaled anticholinergics | Ipratropium bromide | R01AX03 | 2246084, 2246083, 2163705, 2163713, 2240508, 2240072 |
|  |  | R03BB01 | 2126222, 2243827, 2231494, 731439, 576158, 2247686, 824216, 2026759, 1950681, 2239131, 2216221, 2210479, 2231785, 2236934, 2236935, 2237134, 2237135, 2239627, 2231135, 2231136, 2231245, 2231244, 2097141, 2097176, 2097168 |
|  | Ipratropium bromide, fenoterol | R03AK03 | 02148633 |
|  | Tiotropium bromide | R03BB04 | 02246793 |
| Other beta-agonists | Epinephrine | R03AA01 | 2017555, 466417, 525103, 1927582 |
|  | Ephedrine | R03CA02 | 2237085, 2229698, 2100231, 2100258, 2243148, 2236722, 2229678, 2219743, 2012111, 2229711, 38121, 2242961, 876534, 893323, 893331, 438847, 2242639, 2126419, 2126400 |
|  | Isoprenaline | R03AB02 | 2017652 |
|  | Orciprenaline | R03AB03 | 1923870, 1928449, 2017660, 254134, 3859 |
| Other corticosteroids | Cortisone | H02AB10 | 280437, 16241, 16446, 16438 |
|  | Triamcinolone | H02AB08 | 2194090, 15016, 15024, 2194082 |
|  | Prednisone | H02AB07 | 610623, 598194, 550957, 312770, 252417, 210188, 868426, 868434, 868442, 21695, 232378, 607517, 508586, 156876, 271373, 271381 |
|  | Prednisolone | H02AB06 | 21679, 2230619, 2152541, 2245532 |
|  | Methylprednisolone | H02AB04 | 1934325, 1934333, 1934341, 30759, 30767, 36129, 30988, 2245406, 2245400, 2245408, 2245407, 2241229, 2231893, 2231894, 2231895, 2232750, 2232748, 2063727, 2063697, 2063719, 2063700, 36137, 2230210, 2230211, 30678, 30651, 30643 |
|  | Betamathasone | H02AB01 | 2237835, 36366, 2063190, 176834, 28096, 28185 |
|  | Hydrocortisone | H02AB09 | 888222, 888230, 888206, 888214, 30910, 30929, 872520, 872539, 878618, 878626, 30635, 30600, 30619, 30627 |
|  | Dexamathasone | H02AB02 | 2261081, 2250055, 213624, 16462, 354309, 716715, 874582, 1977547, 664227, 2204274, 2204266, 295094, 285471, 489158, 2239534, 732893, 732885, 2260301, 2237044, 2260298, 2237046, 2237045, 1946897, 1964976, 1964968, 1964070, 2279363, 783900, 751863, 2311267, 2240687, 2240685, 2240684 |
| Other xanthines | Theophylline, combination | R03DA54 | 545090, 476374, 334510, 356123, 792942, 721301, 317225, 828718, 640093, 828726, 828742, 307548 |
| Other anti-allergic agents | Levocabastine | R01AC02 | 2020017 |
|  | Ketotifen | R06AX17 | 2221330, 2176084, 2230730, 2218305, 2231680, 2231679, 600784, 577308 |
