# Supplementary material for: Trends in Asthma-Related Direct Medical Costs from 2002 to 2007 in British Columbia, Canada: A Population Based-Cohort Study
Source: PLoS One. 2012 Dec 5;7(12):e50949. doi: 10.1371/journal.pone.0050949 (PMC3515523; doi:10.1371/journal.pone.0050949)
Supplement: Table S2 — Exclusion of childhood and obstructive lung diseases among patients younger than 5 or older than 55 years old, International Classification of Diseases – Ninth Revision (ICD-9) and Tenth Revision (ICD-10) codes selected in the Discharge Abstracts Database (DAD) (DOCX) [file pone.0050949.s002.docx]

**Appendix Table 2:** Exclusion of childhood and obstructive lung diseases among patients younger than 5 or older than 55 years old, International Classification of Diseases – Ninth Revision (ICD-9) and Tenth Revision (ICD-10) codes selected in the Discharge Abstracts Database (DAD)

| **Condition** | **ICD-9 code** | **ICD-10 code** |
| --- | --- | --- |
| [Chronic] bronchitis | 491.x | J40, J41, J42 |
| Emphysema | 492.x | J43 |
| Bronchiectasis | 494.x | J47 |
| Chronic airway obstruction not elsewhere classified | 496.x | J44 |
