# Supplementary material for: Trends in Asthma-Related Direct Medical Costs from 2002 to 2007 in British Columbia, Canada: A Population Based-Cohort Study
Source: PLoS One. 2012 Dec 5;7(12):e50949. doi: 10.1371/journal.pone.0050949 (PMC3515523; doi:10.1371/journal.pone.0050949)
Supplement: Table S3 — Physician visits for asthma and asthma-related diagnoses used for the ‘broad definition’ of asthma-related resource use, International Classification of Diseases – Ninth Revision (ICD-9) codes selected in the Medical Service Plan (MSP) database (DOCX) [file pone.0050949.s003.docx]

**Appendix Table 3:** Physician visits for asthma and asthma-related diagnoses used for the ‘broad definition’ of asthma-related resource use, International Classification of Diseases – Ninth Revision (ICD-9) codes selected in the Medical Service Plan (MSP) database

| **ICD-9 code** | **Name** |
| --- | --- |
| 32 | Allergy injection |
| 460 | Acute nasopharyngitis |
| 465 | Acute upper respiratory tract infection |
| 466 | Acute bronchitis or bronchiolitis |
| 472 | Chronic pharyngitis/nasopharyngitis |
| 477 | Allergic rhinitis |
| 480 | Viral pneumonia |
| 481 | Pneumococcal pneumonia |
| 482 | Other bacterial pneumonia |
| 485 | Bronchopneumonia |
| 487 | Influenza |
| 490 | Bronchitis—other |
| 491 | Chronic bronchitis |
| 492 | Emphysema |
| 493 | Asthma |
| 494 | Bronchiectasis |
| 496 | Chronic airway obstruction |
